# Metagenomic Information Recovery from Human Stool Samples Is Influenced by Sequencing Depth and Profiling Method

**DOI:** 10.3390/genes11111380

**Published:** 2020-11-21

**Authors:** Tasha M. Santiago-Rodriguez, Aaron Garoutte, Emmase Adams, Waleed Nasser, Matthew C. Ross, Alex La Reau, Zachariah Henseler, Tonya Ward, Dan Knights, Joseph F. Petrosino, Emily B. Hollister

**Affiliations:** 1 Diversigen Inc., Houston, TX 77021, USA; agaroutte@diversigen.com (A.G.); eadams@diversigen.com (E.A.); waleednass@gmail.com (W.N.); jpetrosi@bcm.edu (J.F.P.); ehollister@diversigen.com (E.B.H.); 2 Alkek Center for Metagenomics and Microbiome Research, Baylor College of Medicine, Houston, TX 77030, USA; mcross@bcm.edu; 3 Department of Molecular Virology and Microbiology, Baylor College of Medicine, Houston, TX 77030, USA; 4 Diversigen Inc., Saint Paul, MN 55112, USA; alareau@diversigen.com (A.L.R.); zhenseler@diversigen.com (Z.H.); tward@diversigen.com (T.W.); dknights@diversigen.com (D.K.); 5 Department of Computer Science and Engineering, University of Minnesota, Minneapolis, MN 55455, USA; 6 Biotechnology Institute, College of Biological Sciences, University of Minnesota, Minneapolis, MN 55455, USA

**Keywords:** alignment, marker gene, microbiome, shallow sequencing, shotgun metagenomic sequencing, virome

## Abstract

Sequencing of the 16S rRNA gene (16S) has long been a go-to method for microbiome characterization due to its accessibility and lower cost compared to shotgun metagenomic sequencing (SMS). However, 16S sequencing rarely provides species-level resolution and cannot provide direct assessment of other taxa (e.g., viruses and fungi) or functional gene content. Shallow shotgun metagenomic sequencing (SSMS) has emerged as an approach to bridge the gap between 16S sequencing and deep metagenomic sequencing. SSMS is cost-competitive with 16S sequencing, while also providing species-level resolution and functional gene content insights. In the present study, we evaluated the effects of sequencing depth on marker gene-mapping- and alignment-based annotation of bacteria in healthy human stool samples. The number of identified taxa decreased with lower sequencing depths, particularly with the marker gene-mapping-based approach. Other annotations, including viruses and pathways, also showed a depth-dependent effect on feature recovery. These results refine the understanding of the suitability and shortcomings of SSMS, as well as annotation tools for metagenomic analyses in human stool samples. Results may also translate to other sample types and may open the opportunity to explore the effect of sequencing depth and annotation method.

## 1. Introduction

Sequencing-based studies are commonly employed to identify patterns in the microbiome and cause–effect relationships of microorganisms with their host and environment [1,2]. Although both 16S rRNA gene (16S) sequencing and shotgun metagenomic sequencing (SMS) can be utilized for this purpose, traditional SMS is often more cost-prohibitive than 16S sequencing and typically requires substantially greater computational resources to process [3]. For these reasons, 16S sequencing is often the preferred option to acquire bacterial diversity and taxonomy information in a time- and cost-efficient manner. However, species-level resolution is rarely provided by 16S sequencing, as only one or two variable regions are usually sequenced. In addition, partial 16S sequence reads may not provide the resolution needed to differentiate species that share a high degree of homology across the 16S rRNA gene (e.g., *Escherichia*/*Shigella* and *Staphylococcus* spp.) [4]. Although groundbreaking discoveries have been made using 16S-based data, species-level resolution has become increasingly important for translational microbiology purposes [5,6]. In addition, 16S sequencing does not allow for the identification of viruses, fungi, and other small eukaryotes or the direct identification of pathway information. Although pathway information can be imputed from 16S data, SMS data provide more reliable and complete information that can be used for downstream analyses [7,8,9].

An intrinsic challenge in the application of SMS for microbiome research is determining the sequencing depth necessary for meaningful taxonomic and functional comparisons. A limited number of studies have evaluated the sequencing depth needed to identify important taxonomic and functional signals from SMS data [3,10]. These studies have shown that a sequencing depth of 0.5 M sequences per sample is sufficient to capture taxonomic and functional signals on par with those obtained with sequencing depths > 100 M sequences [3,10]. Other studies using reference materials have also found that taxonomic classification does not necessarily improve beyond 60 M paired sequences, and classification of eukaryotic communities can be estimated with 0.5 M reads [11,12]. Shallow SMS (SSMS) has recently emerged as a cost-effective and information-rich alternative to SMS and 16S sequencing [3]. While SSMS results in a lower number of sequences, it poses several advantages compared to 16S sequencing, including (i) species-level resolution; (ii) direct functional characterization; (iii) identification of viruses, fungi, and other small eukaryotes; (iv) lack of amplification biases associated with 16S sequencing, which can skew community representation; (v) increased cost-efficiency relative to deep SMS, particularly for large-scale studies; and (vi) potentially reduced computational costs relative to deep SMS.

In addition to the sequencing depth, the annotation approach may exert an effect on diversity and taxonomic profiles. Particularly, application of marker gene-mapping- and alignment-based methods for taxonomic classification may influence results. Marker gene-mapping-based tools, such as Metagenomic Phylogenetic Analyses v.2.0 (MetaPhlAn2), infer the presence and read coverage of clade-specific markers to estimate relative abundance of microbial species [13]. On the other hand, alignment-based tools, such as BURST, work as high-speed pairwise sequence aligners and may specialize in aligning short reads against reference databases [14]. The effect of both marker gene-mapping- and alignment-based approaches on metagenomic datasets has not been widely determined in association with sequencing depth.

Taxonomic classification is often restricted to bacteria in microbiome studies [15]. However, viruses also comprise a less studied but important fraction of the microbiome known as the virome [16]. Virome studies often involve the amplification of the virus signal by applying several physical, molecular, and computational methods, including virus separation from the host (e.g., human, animal, and plants) and bacterial cells using cesium chloride (CsCl) gradient ultracentrifugation, DNAse and RNAse treatment prior to nucleic acid extraction, amplification of viral nucleic acids using random or targeted amplification, and in silico removal of host DNA post-sequencing [17,18,19,20]. Despite variation in upstream sample processing across laboratories and research groups, viral taxonomic profiles from SMS data can often be useful and readily available to gather a global understanding of viral community composition. This is particularly true for DNA viruses (when no a priori RNA extraction and cDNA conversion has been applied), which tend to be present at abundances that do not necessarily require viral enrichment step(s) in order to facilitate their detection. Since SMS has the potential to provide viral taxonomic profiles, estimating the SMS depth needed for virome characterization is also of importance.

Here, the main aims of our study were to augment the current understanding of the effect of SMS depths and annotation method (marker gene-mapping- vs. alignment-based) on the detection of bacterial genera in comparison to 16S information, and on the detection of bacterial species, as well as the effect of SMS depth on pathway identification and viral communities, using healthy human stool samples as proxies of the gut microbiome.

## 2. Materials and Methods

### 2.1. Samples Processing for 16S rRNA Sequencing

Ten human stool samples were purchased from Lee Biosolutions (Maryland Heights, MO, USA) and stored at −20 °C until further processing. ATCC^®^ mock community MSA1001, comprised of DNA from 10 organisms in staggered concentrations (ranging in relative abundances from 44.78 to 0.04%) was processed in parallel as a control. A no template PCR sample was included as a negative control. For 16S rRNA gene sequencing of the ten stool samples, the V4 hypervariable region was amplified by PCR and sequenced on the MiSeq platform (Illumina; San Diego, CA, USA) using the 2 × 250 bp paired-end protocol following manufacturer’s instructions (Illumina; San Diego, CA, USA). Amplification of the V4 region of the 16S gene was selected in the present study, because it is well-standardized, commonly utilized, and has been shown to capture greater diversity than other variable regions in stool samples [21]. The primers used for amplification contain adapters for sequencing in the Illumina MiSeq platform, which also contains single-end barcodes, allowing pooling and direct sequencing of PCR products [22]. The read pairs were demultiplexed followed by merging using USEARCH v7.0.1090 [23], allowing zero mismatches and a minimum overlap of 50 bases. Merged reads were trimmed using Trimmomatic (version 0.39) to perform adapter trimming, quality trimming, and filtering (SLIDINGWINDOW:4:20 MINLEN:75) at the first base with Q5 [24]. In addition, a quality filter was applied to the resulting merged reads and reads containing above 0.05 expected errors were discarded. The 16S rRNA gene sequences were clustered into operational taxonomic units (OTUs) at a similarity cutoff value of 97% using the UPARSE algorithm [25]. OTUs were mapped to an optimized version of the SILVA (v132) database containing only the 16S v4 region from 6,087,080 rRNA gene sequences to determine taxonomies. Abundances were recovered by mapping the demultiplexed reads to the UPARSE OTUs. All downstream analyses including α-diversity, β-diversity, and taxonomic analyses were performed using in-house data visualization tools. For comparison with SMS data, OTUs were collapsed to genus level.

### 2.2. Shotgun Metagenomic Library Preparation, Sequencing, and Data Quality 

Genomic DNA from the ten stool samples described above, as well as ATCC^®^ mock community MSA1001, were prepared for sequencing using the Nextera DNA Flex library preparation kit (Illumina; San Diego, CA, USA) with Nextera Index Kit (Illumina; San Diego, CA, USA). Library size estimation and quantification were determined with the fragment analyzer (Advanced Analytical Technologies, Inc., Ankeny, IA, USA) electrophoresis system. Sequencing was performed on a NextSeq using the 2 × 150 bp paired-end protocol following manufacturer’s instructions (Illumina; San Diego, CA, USA). After sequencing, Trimmomatic (version 0.39) was used as described above [24]. After quality trimming and filtering, host filtering was performed by aligning reads to a human reference genome (GRCh38; GCA_000001405.15) that was pulled from Bowtie2 (version 2.3.4.1) with default parameters [26]. Unmapped reads were retained for downstream analysis, including taxonomic, pathway, and viral profiling.

### 2.3. Random Subsampling of Sequences

After quality check (QC), shotgun metagenomic sequences were subsampled using the fastq-sample command from the fastq-tools suite downloaded from https://homes.cs.washington.edu/~dcjones/fastq-tools/. Reads were subsampled at the following thresholds: 5 Gb (16.67 M reads), 3 Gb (10.00 M reads), 1 Gb (3.00 M reads), 0.75 Gb (2.50 M reads), 0.5 Gb (1.65 M reads), 0.25 Gb (0.85 M reads), and 0.1 Gb (0.34 M reads).

### 2.4. Taxonomic and Pathway Annotations

After subsampling the reads, bacterial taxonomic annotations were performed using MetaPhlAn2 (v2.7.7) [13], which uses clade-specific markers that provide bacterial, archaeal, viral, and eukaryotic quantification at the species level using default parameters for reported relative abundances. This version of the MetaPhlAn database was developed using 16,904 reference genomes. Since the bacterial component was the most abundant, only bacteria were considered, and data were re-normalized prior to downstream analysis. DNA sequences were also aligned to the Venti database (originally developed at CoreBiome), a curated proprietary database containing 19,840 complete and/or high-quality draft bacterial genomes from RefSeq. Alignments were made at 97% identity against all reference genomes. Every input sequence was compared to every reference sequence in the Venti database using fully gapped alignment with the BURST mapping algorithm [14]. Ties were broken by minimizing the overall number of unique genome hits. For taxonomy assignment, each input sequence was assigned the lowest common ancestor that was consistent across at least 80% of all reference sequences tied for best hit. The number of counts for each genome was normalized to genome length, and genomes accounting for less than one millionth of all species-level hits and those with less than 0.01% of their unique genome regions covered (and <1% of the whole genome) were discarded. Samples with fewer than 10,000 sequences were also discarded. Count data were converted to relative abundance for each sample. Both MetaPhlAn2 and BURST data were further filtered to remove taxa present in relative abundances <0.01% using the script filter_otus_from_otu_table.py in the Quantitative Insights into Microbial Ecology (QIIME) platform [27]. The normalized and filtered table was used for all downstream analyses.

Viral profiles from the SMS data, subsampled to 5 Gb (16.67 M reads), 3 Gb (10.00 M reads), 1 Gb (3.00 M reads), 0.75 Gb (2.50 M reads), 0.50 (1.65 M reads), 0.25 Gb (0.85 M reads), and 0.1 Gb (0.34 M reads), were obtained using VirMap [28]. VirMap merges nucleotide and protein information to assign taxonomy independently from genome coverage or read overlap. Relative abundances of selected viruses were acquired by dividing the number of counts of the virus of interest per sequencing depth divided by the genome size in bp.

Pathway profiling was performed using HUMAnN2 v0.11.1 with default parameters [29]. Pathway searches were performed against UniProt and MetaCyc50 [30,31]. Unclassified and unmapped categories were removed, and data were re-normalized for further analyses.

### 2.5. Statistical Analyses

Statistical analyses were performed in RStudio version 1.1.456. For α-diversity comparisons, the Friedman Rank test was used to determine statistical differences among sequencing depths, as repeated measure was considered, and the Nemenyi post hoc test was further applied for sequencing depth comparisons. Permutational multivariate analysis of variance (PERMANOVA) was performed using proprietary tools to visualize differences based on weighted Bray–Curtis distances. Distance values were then visualized using Principal Coordinate Analysis (PCoA) plots. Nonparametric permutation tests were employed to test for statistical significance amongst sequencing depths and taxonomy and pathways relative abundances. Briefly, comparisons of genera or species across the various sequencing depths were performed using Kruskal–Wallis in QIIME using the group_significance_analyses.py script, with default parameters. The group_significance.py script compares each feature (e.g., OTU, species, gene family) to see if it is differentially abundant based on the groupings of interest, as defined in the metadata file. The script will group together samples that have the same value in the mapping file under the header passed with the -c option. The script output includes unadjusted *p*-values, and false discovery rate (FDR) values. Correlation plots were generated using the function ggscatter () in RStudio with the addition of correlation coefficient and *p*-value to indicate significance.

### 2.6. Data Availability

The sequences generated and analyzed during the current study are available in the NCBI repository under accession number PRJNA676159.

## 3. Results

### 3.1. Bacterial Richness, but Not Evenness or β-Diversity, Is Affected by Sequencing Depth When Using Marker Gene-Mapping- and Alignment-Based Approaches

**Figure 1:**
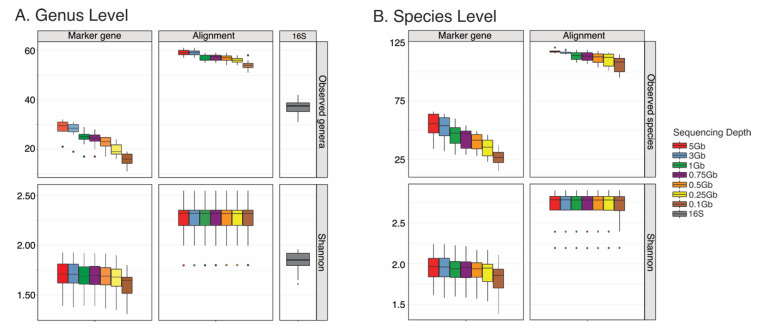
Boxplots of the α-diversity of ten stool samples from healthy subjects at various sequencing depths, including 5 Gb (16.67 M reads), 3 Gb (10.00 M reads), 1 Gb (3.00 M reads), 0.75 Gb 2.50 M reads), 0.5 Gb (1.65 M reads), 0.25 Gb (0.85 M reads), and 0.1 Gb (0.34 M reads). The 16S sequencing analysis at the genus level was performed for comparison. (**A**) Number of observed genera and Shannon diversity at the genus level for the marker gene-mapping and alignment methods. (**B**) Number of observed species and Shannon diversity at the species level for the marker gene-mapping and alignment methods. Outliers are shown.

A total of 10 healthy human stool samples were included in the present study. After QC, a total average number of 25,491,462 ± 5,449,536 sequences were analyzed (Supplementary Table S1). SMS data were subsampled to 5 Gb (16.67 M reads), 3 Gb (10.00 M reads), 1 Gb (3.00 M reads), 0.75 Gb (2.50 M reads), 0.5 Gb (1.65 M reads), 0.25 Gb (0.85 M reads), and 0.1 Gb (0.34 M reads) and annotated using MetaPhlAn v.2.0 [13], as well as BURST to assess the potential effects of marker gene-mapping- and alignment-based approaches on bacterial taxonomic classification, respectively [14]. In addition, we performed 16S sequencing for comparison at the genus level. Prior to further analyses, data were filtered to remove bacterial genera and species with relative abundances < 0.01% to decrease potential false positives. This was performed separately for both stool and mock community samples. α-diversity at the genus level was determined at the various mentioned sequencing depths (Figure 1).

Results show a slightly lower number of observed genera using SMS and a marker gene-mapping-based approach at 5 Gb compared to 16S sequencing (Figure 1A) (Supplementary Dataset 1), although this difference was not statistically significant (*p*-value = 0.96) (Supplementary Dataset 2). Observed genera significantly decreased at 0.5 Gb compared to 5 Gb (*p*-value = 0.020) when data were annotated using the marker gene-mapping-based approach (Supplementary Dataset 2). Interestingly, higher numbers of observed genera were noted at all sequencing depths when using the alignment approach compared to 16S sequencing at similar depths (Figure 1A) (Supplementary Dataset 1). Differences between the alignment vs. the 16S methods were determined using a Friedman rank sum test and were statistically significant at 5 Gb (*p*-value = 1.40 × 10^−7^), 3 Gb (*p*-value = 4.20 × 10^−7^), 1 Gb (*p*-value = 8.60 × 10^−4^), and 0.75 Gb (*p*-value = 2.65 × 10^−3^). Observed genera significantly decreased at 0.25 Gb (*p*-value = 3.79 × 10^−3^) compared to 5 Gb when data were annotated using the alignment-based approach. Shannon diversity at the genus level did not significantly decrease at any of the sequencing depths tested when using the marker gene-mapping-based- and alignment-based approaches (Figure 1A and Supplementary Dataset 1).

α-diversity analyses also included observed species and Shannon diversity at the species level using Friedman rank sum test (Figure 1B). While the number of observed species was not significantly different between the marker gene-mapping-based approach (5 Gb) and 16S sequencing (*p*-value = 0.99), the number of observed species identified at 5 Gb (*p*-value = 1.10 × 10^−8^), 3 Gb (*p*-value = 2.00 × 10^−6^), 1 Gb (*p*-value = 7.00 × 10^−4^), and 0.75 Gb (*p*-value = 0.002) when using the alignment-based approach was significantly higher than those observed when using 16S sequencing. Notably, most of the 16S sequences were unable to be classified at the species level (Supplementary Dataset 3). The number of observed species significantly decreased at 0.5 Gb when using the marker gene-mapping- (*p*-value = 0.009) and alignment-based approaches (*p*-value = 0.012) compared to 5 Gb (Supplementary Dataset 2).

**Figure 2:**
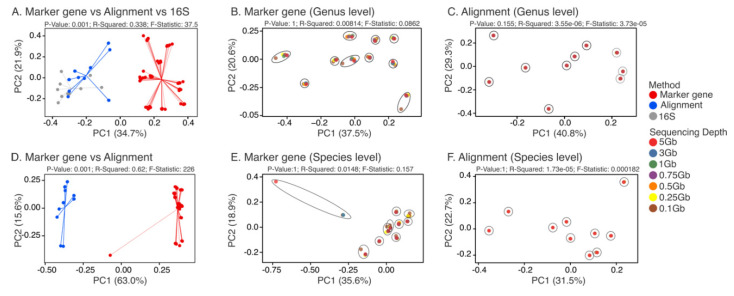
PCoA plots of the β-diversity (weighted Bray–Curtis distances) of ten stool samples from healthy subjects at various sequencing depths including 5 Gb (16.67 M reads), 3 Gb (10.00 M reads), 1 Gb (3.00 M reads), 0.75 Gb (2.50 M reads), 0.5 Gb (1.65 M reads), 0.25 Gb (0.85 M reads), and 0.1 Gb (0.34 M reads). The 16S rRNA (16S) V4 hypervariable region sequencing analysis at the genus level was performed for comparison. (**A**) SMS data annotated using the marker gene mapping and alignment methods, in comparison with the 16S sequencing information described above at the genus level. (**B**) SMS data annotated using the marker gene-mapping method at the genus level. (**C**) SMS data annotated using the alignment method at the genus level. (**D**) SMS data annotated using the marker gene mapping and alignment methods at the species level. (**E**) SMS data annotated using the marker gene-mapping method at the species level. (**F**) SMS data annotated using the alignment method at the species level. Clustering of data by subjects is highlighted using circles or ellipses.

β-diversity (Bray–Curtis weighted dissimilarity distances) of the stool samples at the genus level showed separation of samples based on method (marker gene-mapping vs. alignment vs. 16S sequencing), with the 16S sequencing data clustering closer to the alignment-based data (PERMANOVA; *p*-value = 0.001; R-Squared = 0.338; F-Statistic = 37.5) (Figure 2A). PCoA plots of the marker gene-mapping-based Bray–Curtis distances at the genus level showed clustering by subject rather than sequencing depth (PERMANOVA; *p*-value = 1; R-Squared = 0.00814; F-Statistic = 0.0862) (Figure 2B). Interestingly, subject-specific effects were noted, as marker gene-mapping-based data from some subjects at 0.1 Gb did not cluster closely to their counterpart profiles generated at greater sequencing depths (Figure 2B). Similarly, PCoA plots of the alignment-based Bray–Curtis distances at the genus level showed clustering by subject (PERMANOVA; *p*-value = 0.155; R-Squared = 3.55 × 10^−6^; F-Statistic = 3.73 × 10^−5^) (Figure 2C). Bray–Curtis weighted dissimilarity distances of the stool samples at the species level showed separation of the data based on the method used (marker gene mapping vs. alignment) (PERMANOVA; *p*-value = 0.001; R-Squared = 0.62; F-Statistic = 226) (Figure 2D). Plotting of the marker gene-mapping- (PERMANOVA; *p*-value = 1; R-Squared = 0.0148; F-Statistic = 0.157) (Figure 2E) and alignment-based data (PERMANOVA; *p*-value = 1; R-Squared = 1.73 × 10^−5^; F-Statistic = 0.000182) (Figure 2F) at the species level showed clustering by subject.

### 3.2. Identification of Certain Bacterial Genera and Species Is Affected by SMS Sequencing Depth When Using a Marker Gene-Mapping-Based Approach, but Not an Alignment-Based Approach

**Figure 3:**
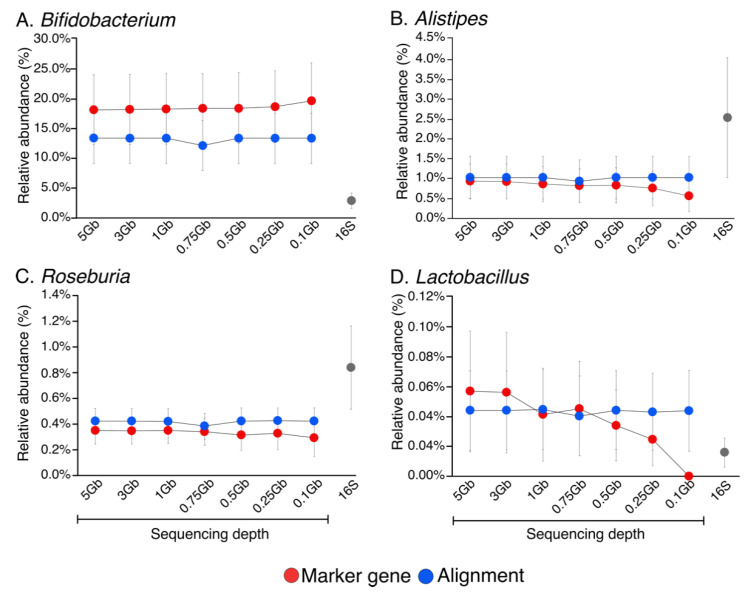
Line charts of the average relative abundances (%) (genus level) of selected stool taxa based upon SMS data annotated using marker gene-mapping- and alignment-based methods. Data were annotated at various sequencing depths including 5 Gb (16.67 M reads), 3 Gb (10.00 M reads), 1 Gb (3.00 M reads), 0.75 Gb (2.50 M reads), 0.5 Gb (1.65 M reads), 0.25 Gb (0.85 M reads), and 0.1 Gb (0.35 M reads). The 16S sequencing data were included for comparison. (**A**) *Bifidobacterium* SMS data annotated using marker gene-mapping- and alignment-based methods. (**B**) *Alistipes* SMS data annotated using marker gene-mapping- and alignment-based methods. (**C**) *Roseburia* SMS data annotated using marker gene-mapping- and alignment-based methods. (**D**) *Lactobacillus* SMS data annotated using marker gene-mapping- and alignment-based methods. Standard error is shown by error bars.

The effects of marker gene-mapping- and alignment-based tools at various SMS sequencing depths, as well as 16S sequencing, to the relative abundances of bacterial genera were determined (Supplementary Dataset 3 and Supplementary Dataset 4). The average relative abundances of common gut commensals typically varying in abundance in human stool, including *Bifidobacterium* (Figure 3A), *Alistipes* (Figure 3B), *Roseburia* (Figure 3C), and *Lactobacillus* (Figure 3D) were plotted. Relative abundances of these commensals were relatively consistent across sequencing depths, with the exception of *Lactobacillus*, when using the marker gene-mapping- versus alignment-based approaches (Supplementary Dataset 3 and Supplementary Dataset 4). *Lactobacillus*, which is present at relatively low abundance, was rarely detectable when using the marker gene-mapping-based approach at a sequencing depth < 0.25 Gb (Figure 3D). Notably, each commensal exhibited varying abundances depending on the sequencing depth and annotation approach (Supplementary Dataset 4). In addition, marker gene-mapping, alignment, and 16S approaches each captured distinctive taxa (Supplementary Dataset 3 and Supplementary Dataset 4).

**Figure 4:**
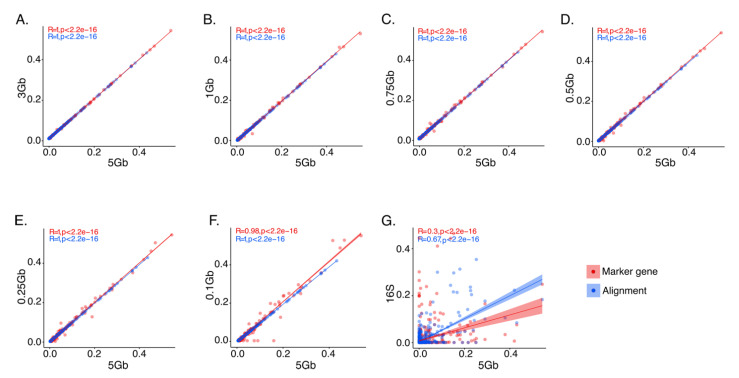
Correlation plots of the relative abundances of bacterial genera annotated using marker gene-mapping and alignment methods. (**A**) Correlations performed with data obtained at 5 Gb (16.67 M reads) vs. 3 Gb (10.00 M reads). (**B**) Correlations performed with data obtained at 5 Gb vs. 1 Gb (3.00 M reads). (**C**) Correlations performed with data obtained at 5 Gb vs. 0.75 Gb (2.50 M reads). (**D**) Correlations performed with data obtained at 5 Gb vs. 0.5 Gb (1.65 M reads). (**E**) Correlations performed with data obtained at 5 Gb vs. 0.25 (0.85 M reads). (**F**) Correlations performed with data obtained at 5 Gb vs. 0.1 Gb (0.35 M reads). (**G**) Correlations performed with data obtained at 5 Gb vs. 16S sequencing data.

Correlations of all bacterial genera across the various SMS depths using the marker gene-mapping- and alignment-based approaches, as well as 16S, were performed to demonstrate the extent of signal variation relative to depth and annotation approach. Data obtained at 5 Gb from both the marker gene-mapping- and alignment-based approaches showed strong, statistically significant correlations at 3 Gb (Figure 4A), 1 Gb (Figure 4B), 0.75 Gb (Figure 4C), 0.5 Gb (Figure 4D), and 0.25 Gb (Figure 4E) (R =1; *p*-value < 2.2 × 10^−16^). Strong, statically significant correlations were also noted with the alignment-based approach at 0.1 Gb (Figure 4F) (R = 1; *p*-value < 2.2 × 10^−16^), but these were slightly decreased when using the marker gene-mapping-based approach (R = 0.98; *p*-value < 2.2 × 10^−16^). Moderate (R = 0.67; *p*-value < 2.2 × 10^−16^) and weak (R = 0.3; *p*-value < 2.2 × 10^−16^) correlations were noted between the alignment- and marker gene-mapping-based information, with 16S sequencing, respectively (Figure 4G).

The effect of SMS sequencing and the annotation method used (marker gene-mapping- vs. alignment-based) for bacterial species identification was determined (Supplementary Dataset 5 and Supplementary Dataset 6). Results show that sequencing depth does not affect the detection of bacterial species annotated using the alignment-based approach, but it does affect the detection of certain bacterial species when using a marker gene-mapping-based approach (Supplementary Dataset 6 and Table 1). To test this, the group_significance.py script using Kruskal–Wallis as the test was applied to see if each taxon was differentially abundant at the various sequencing depths. For instance, *Clostridium spiroforme* (FDR-adjusted *p*-value = 2.16 × 10^−2^) and *Anaerotruncus colihomnis* (FDR-adjusted *p*-value = 3.26× 10^−2^) were not detected at sequencing depths < 3 Gb when using a marker gene-mapping-based approach. Additional bacterial species not detected at sequencing depths lower than 0.5 Gb and 0.25 Gb, as in the case of *Lactococcus lactis* (FDR-adjusted *p*-value = 2.16 × 10^−2^) and *Lachnospiraceae bacterium* 3 1 46FAA (FDR-adjusted *p*-value = 3.55 × 10^−2^), respectively (Table 1).

### 3.3. Assessing the Accuracy of Marker Gene-Mapping- and Alignment-Based Approaches Using Reference Materials

To assess the accuracy of both the marker gene-mapping- and alignment-based methods, mock community data (ATCC^®^ MSA1001) were annotated at the various sequencing depths tested. At the genus level, both annotation methods identified all 10 expected taxa. While no false positives were identified with the marker gene-mapping-based approach, the alignment-based approach incorrectly identified *Shigella*, which was not present in the mock community. The marker gene-mapping-based approach identified all 10 bacterial genera at 5 Gb in relative abundances similar to those expected (Supplementary Dataset 7 and Supplementary Figure S1A). However, *Bifidobacterium, Deinococcus*, and *Enterococcus*, expected to be present in relative abundances of 0.04%, were not identified at a sequencing depth < 0.5 Gb. The alignment-based approach underestimated the expected abundance of *Escherichia*, but the remaining bacterial genera were identified at all sequencing depths in relative abundances compared to the expected relative abundances (Supplementary Dataset 7 and Supplementary Figure S1B).

At the species level, all 10 of the expected taxa were identified when using the marker gene-mapping-based method at 5 Gb. The *Deinococcus radiodurans* signal was lost at a sequencing depth of <0.5 Gb, while *Bifidobacterium adolescentis* and *Enterococcus faecalis* signals were lost at sequencing depths < 0.25 Gb when using the marker gene-mapping-based approach (Supplementary Figure S1C and Supplementary Dataset 7). The alignment-based approach captured all 10 of the expected taxa across the various sequencing depths but underestimated the relative abundance of *B. adolescentis* and *Escherichia coli* (Supplementary Figure S1D). Interestingly, approximately 55.4% of the sequences in the mock community were not classified (Unclassified), while 2.5% and 0.1% of the sequences were attributed to *Bacillus cereus* and *Staphylococcus* sp. HMSC055B03, respectively (Supplementary Dataset 7).

### 3.4. Sequencing Depth Affects Virus Identification from Metagenomic Samples and Is Subject-Specific

**Figure 5:**
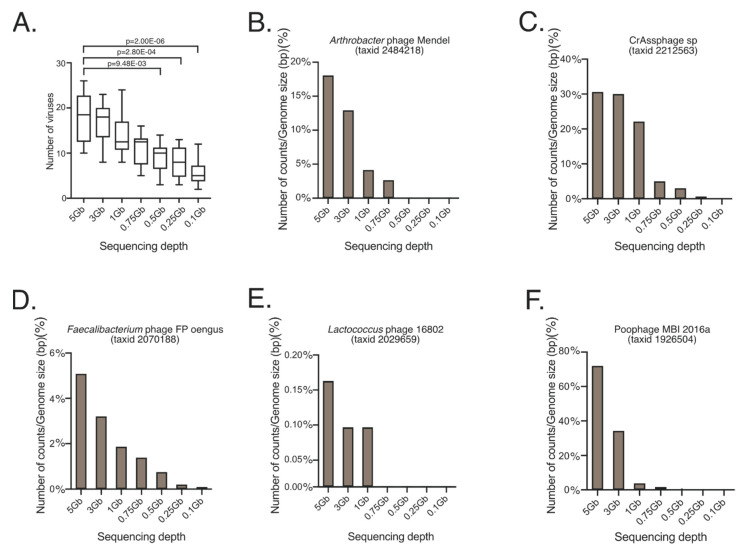
Viruses identified in healthy individuals at various sequencing depths including 5 Gb (16.67 M reads), 3 Gb (10.00 M reads), 1 Gb (3.00 M reads), 0.75 Gb (2.50 M reads), 0.5 Gb (1.65 M reads), 0.25 Gb (0.85 M reads), and 0.1 Gb (0.35 M reads). (**A**) Boxplots of the total number of viruses identified in ten healthy individuals at the various sequencing depths described. Figure also shows barplots of normalized counts (%), consisting of total read counts per virus at the various sequencing depths, divided by the genome size (bp). (**B**) *Arthrobacter* phage Mendel (taxid 2484218) total read counts/genome size (bp) (%). (**C**) CrAssphage (taxid 2212563) total read counts/genome size (bp) (%). (**D**) *Faecalibacterium* phage FP oengus (taxid 2070188) total read counts/genome size (bp) (%). (**E**) *Lactococcus* phage 16802 (taxid 2029659) total read counts/genome size (bp) (%). (**F**) Poophage MBI 2015a (taxid 1926504) total read counts/genome size (bp) (%).

SMS data at 5 Gb, 3 Gb, 1 Gb, 0.75 Gb, 0.5 Gb, 0.25 Gb, and 0.1 Gb were processed for virus taxonomic characterization using VirMap [28]. The total number of identified viruses decreased significantly at a sequencing depth below 0.5 Gb (*p*-value = 9.0 × 10^−4^) (Figure 5A). The stochasticity of sampling sequences with low counts was evident with the virome dataset analyzed. This limited the ability to fully understand the effect of SMS depth relative to viral detection; however, data could still be used as a proof-of-concept. For this, virus abundances from individual, healthy subjects were normalized by dividing the total counts by the virus genome size (bp). Percentages of normalized counts were then plotted. Values for *Arthrobacter* phage Mendel (taxid 2484218) (Figure 5B), CrAssphage (taxid 2212563) (Figure 5C), *Faecalibacterium* phage FP cengus (taxid 2070188) (Figure 5D), *Lactococcus* phage 16802 (taxid 2029659) (Figure 5E), and Poophage MBI 2016a (taxid 1926504) (Figure 5F) were plotted. Although not all the viruses shown in Figure 5B–F were identified in all the subjects, these results suggest that 5 Gb recovered more viruses than lower sequencing depths (Figure 5A), but the implications of these findings should be considered in a subject-to-subject and virus-per-virus scenario (Supplementary Dataset 8).

### 3.5. Pathway Relative Abundances Are Dependent on Sequencing Depth and Are Category-Specific

**Figure 6:**
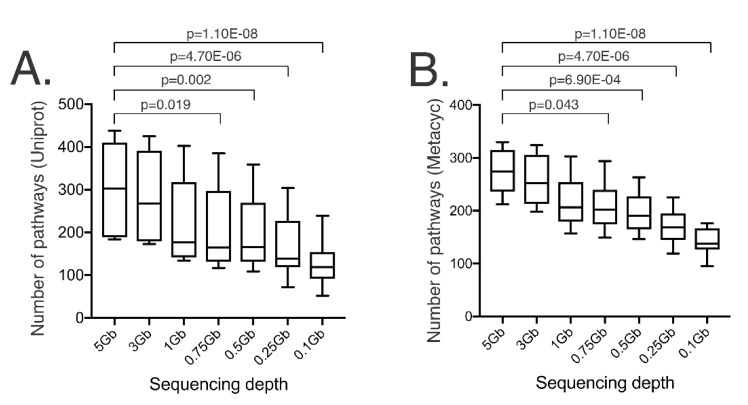
Boxplots of the total number of functional pathways identified in ten stool samples across various sequencing depths including 5 Gb (16.67 M reads), 3 Gb (10.00 M reads), 1 Gb (3.00 M reads), 0.75 Gb (2.50 M reads), 0.5 Gb (1.65 M reads), 0.25 Gb (0.85 M reads), and 0.1 Gb (0.34 M reads). (**A**) Number of UniProt pathways across the various sequencing depths. (**B**) Number of MetaCyc pathways across the various sequencing depths.

Pathway information was obtained using HUMAnN2, with UniProt and MetaCyc databases [29]. HUMAnN2 provides a relatively fast and accurate species-resolved functional profiles by aligning the reads to species pangenomes, performing translated search on unclassified reads, and quantifying gene families and pathways. The total number of pathways identified using UniProt significantly decreased at a sequencing depth below 0.75 Gb (*p*-value = 0.019) (Figure 6A). The pathways identified using UniProt are shown in Supplementary Dataset 9. The relative abundances of a number of pathways identified using UniProt were not significantly different across the various sequencing depths (Supplementary Dataset 10). Similarly, the total number of pathways identified using MetaCyc were significantly reduced at a sequencing depth below 0.75 Gb (*p*-value = 0.043) (Figure 6B). All MetaCyc pathways identified are shown in Supplementary Dataset 11. The number of recovered MetaCyc pathways were significantly affected by sequencing depth (Supplementary Dataset 12). For instance, when looking at the MetaCyc pathway output, L-glutamine biosynthesis pathway was not recovered at a sequencing depth < 0.5 Gb (FDR-adjusted *p*-value = 9.38 × 10^−4^). Other categories, such as the superpathway (which usually have an additional preceding class within the pathway ontology in order to define their biological role) of menaquinol-6 biosynthesis (FDR-adjusted *p*-value = 0.020) and the superpathway of demethylmenaquinol-6 biosynthesis (FDR-adjusted *p*-value = 0.020) were not identified at a sequencing depth < 1 Gb (Supplementary Dataset 12).

## 4. Discussion

The present study aimed to augment current understanding of the effect of both sequencing depth and annotation methods using human stool samples and a mock community with a goal of helping to inform decision making in terms of sequencing method, depth, and annotation tool, particularly for pilot studies and/or studies with high volume samples. Marker gene-mapping- and alignment-based methods for SMS were applied to determine bacterial taxonomic classification and compared with 16S rRNA gene (V4) genus level profiles. Bacterial taxonomic comparison between SMS and 16S at the genus level and at various sequencing depths has not been extensively evaluated. In addition, bacterial species level classification at the various SMS depths was determined using both marker gene-mapping- and alignment-based tools, which have not extensively been evaluated in parallel. The present study also evaluated the effect of SMS depth on the recovery of functional pathways and viral taxa, two categories of microbiome data that are frequently overlooked. Results of the present study build upon previous studies that have sought to assess the effect of sequencing depth with regard to various body sites using k-mer and alignment methods [32]; stool samples and reference materials using marker, k-mer, and alignment methods [11]; stool and food samples, as well as reference materials using k-mer-based methods [33].

Achieving accurate taxonomic and functional resolution is increasingly important when aiming to identify patterns in microbiome studies. However, this is often not achieved when sequencing one or two 16S variable regions, as with amplicon sequencing. In the present study, α-diversity results at the genus level showed lower numbers of genera when using SMS data annotated using the marker gene-mapping-based approach compared to 16S sequencing. This is, in part, a reflection of the availability of a large number of unclassified OTUs at the genus level in 16S databases, as well as the ability of amplicon-based sequencing to capture low-abundance taxa. In addition, that a number of observed genera were not identified using the marker gene-mapping- or the alignment-based approach indicates that reference databases will continue to benefit from the sequencing of additional novel bacterial. The ability to detect low-abundance bacterial genera and species being impaired at shallow sequencing depths when using the marker gene-mapping-based approach suggests that the combination of shallow sequencing and marker gene-mapping-base annotation may not be suitable for studies seeking to characterize low-abundance bacterial taxa, including those that are transient members of the gut microbiome. Notably, the number of bacterial genera and species detected were greater when using the alignment method compared to both the marker gene-mapping-based approach and 16S sequencing. This may be in part due to differing numbers of reference genomes in the interrogated databases, or alignment of reads to closely related taxa. While only one 16S database and variable region were tested, results open the opportunity to evaluate the performance of additional 16S databases, as well as other 16S variable regions in comparison with SMS results. α-diversity and relative abundance results from the selected taxa showed that the alignment-based method may have a greater concordance in signal across sequencing depths and indicate that this approach may be more suitable when using SMS with the caveat of having reads aligned to closely related bacteria, which could result in false positives (Table 2). In addition, these data indicate that, in some cases, 16S sequencing may be more suitable depending on the organism of interest. These data demonstrate that the selection of both sequencing depth and annotation method for taxonomic classification should be carefully evaluated and, ideally, include reference materials to assess performance. Results from reference materials in the present study demonstrated that further filtering of data may be needed to decrease the number of false positives with the caveat of losing taxa present in extremely low relative abundances. This was the case for the alignment-based approach where the selected filtering percentage (i.e., 0.01%) reduced the number of false positives. Results suggest that further filtering may need to be tested accordingly.

SMS also exerts an effect on the lower-abundance, less-characterized aspects of the microbiome. By using viruses as an example, this study demonstrates that the effect of the sequencing depth is more profound when studying the less-studied members of the human microbiome. While the annotation method may also affect virus detection and identification, testing different tools was outside the scope of the present study and has previously been examined in comparison with VirMap [28]. Although not all viruses were identified in similar relative abundances in all subjects, as viromes tend to be personalized, results here provide proof-of-concept data covering various subject-to-subject and virus-to-virus scenarios at various sequencing depths. While we were limited to 5 Gb, it seemed that > 5 Gb may be best suited to capture DNA viruses from SMS data when no a priori isolation/concentration steps for viruses have been applied (Table 2). Results may also translate to other low abundant categories of the microbiome, including potential biomarkers, virulence factors, and antibiotic-resistant genes.

While taxonomic assignments provide key information in microbiome studies, pathway information has the ability to provide insights into the potential role(s) of specific microbial members in maintaining health, promoting disease, and/or be associated/correlated with specific processes. Understanding pathway profiles also represents an opportunity to develop metabolite-based therapeutics, also known as “postbiotics” [34]. Various well-known examples of postbiotics include short-chain fatty acids (SCFAs) [35], taurine [36], and flavonoids [37]. SMS provides insights into the identification of genes associated with the metabolism of some of these specific molecules, but sequencing depth also has the potential to influence information recovery and data interpretation. SSMS may be suitable for a global pathway profile characterization, but it may not be suitable for the characterization of rare pathways, or those that are present at low relative abundances. For this reason, the expected prevalence and abundance of each pathway must be considered to determine the specific sequencing depth needed. Nevertheless, a sequencing depth of approximately 1 Gb in human stool samples appears to stably capture a large proportion of pathways. Future studies need to focus on determining the performance of various pathway annotation tools at various sequencing depths and sample types using appropriate reference materials, as needed (Table 2).

## 5. Conclusions

In this study, we evaluated the combined effects of depth of sequencing and annotation method (i.e., marker gene mapping- vs. alignment-based approaches) on information recovery for SSMS. Sequencing depths > 0.25 Gb and > 0.5 Gb capture α-diversity and genus-level taxonomic abundances similar to those recovered at sequencing depth of 5 Gb when using marker gene-mapping and alignment methods, respectively. Sequencing depths > 0.5 Gb captured bacterial species information at levels analogous to those detected with a sequencing depth of 5 Gb. Pathway recovery at sequencing depths > 0.75 Gb was comparable to that achieved at 5 Gb. Virus profiles were individualized, and signal recovery was impaired at sequencing depths < 5 Gb. Results suggest that sequencing depth and annotation method need to be carefully evaluated depending on the aims of the study.

## Figures and Tables

**Table 1:** Group significance analyses (Kruskal–Wallis) of ten stool samples at various SMS sequencing depths and annotated using a marker gene-mapping- and alignment-based approaches. Table shows the mean relative abundance (%) of bacterial species that are significantly different across the various sequencing depths when considering FDR-adjusted *p*-values (FDR P).

		Sequencing Depth						
**Marker Gene Mapping**	**FDR P**	**5 Gb**	**3 Gb**	**1 Gb**	**0.75 Gb**	**0.5 Gb**	**0.25 Gb**	**0.1 Gb**
*C. spiroforme*	2.16 × 10^−2^	3.05 × 10^−3^	2.55 × 10^−3^	0.00 × 10^0^	0.00 × 10^0^	0.00 × 10^0^	0.00 × 10^0^	0.00 × 10^0^
*L. lactis*	2.16 × 10^−2^	3.72 × 10^−4^	3.25 × 10^−4^	1.80 × 10^−4^	2.37 × 10^−4^	4.95 × 10^−5^	0.00 × 10^0^	0.00 × 10^0^
*Roseburia inulinivorans*	2.69 × 10^−2^	3.43 × 10^−4^	3.19 × 10^−4^	2.13 × 10^−4^	1.80 × 10^−4^	5.65 × 10^−5^	0.00 × 10^0^	0.00 × 10^0^
*L.* bacterium 3 1 46FAA	2.69 × 10^−2^	6.07 × 10^−4^	5.70 × 10^−4^	4.67 × 10^−4^	4.39 × 10^−4^	4.33 × 10^−4^	2.23 × 10^−4^	0.00 × 10^0^
*A. colihominis*	3.26 × 10^−2^	2.84 × 10^−5^	1.24 × 10^−5^	0.00 × 10^0^	0.00 × 10^0^	0.00 × 10^0^	0.00 × 10^0^	0.00 × 10^0^
*Eggerthella lenta*	3.55 × 10^−2^	1.34 × 10^−4^	1.35 × 10^−4^	1.35 × 10^−4^	9.57 × 10^−5^	8.66 × 10^−5^	1.76 × 10^−5^	0.00 × 10^0^
*Clostridium bolteae*	3.55 × 10^−2^	1.11 × 10^−3^	2.22 × 10^−4^	1.13 × 10^−4^	1.37 × 10^−4^	8.32 × 10^−5^	4.48 × 10^−5^	0.00 × 10^0^
*Streptococcus parasanguinis*	3.55 × 10^−2^	7.19 × 10^−4^	6.53 × 10^−4^	5.14 × 10^−4^	4.78 × 10^−4^	3.33 × 10^−4^	2.02 × 10^−4^	0.00 × 10^0^
*Lachnospiraceae* bacterium 7 1 58FAA	3.55 × 10^−2^	2.31 × 10^−4^	2.20 × 10^−4^	1.42 × 10^−4^	1.70 × 10^−4^	1.37 × 10^−4^	6.34 × 10^−5^	0.00 × 10^0^
*Holdemania filiformis*	3.55 × 10^−2^	3.58 × 10^−4^	3.60 × 10^−4^	2.73 × 10^−4^	1.68 × 10^−4^	5.65 × 10^−5^	0.00 × 10^0^	0.00 × 10^0^
*Eubacterium eligens*	3.55 × 10^−2^	1.43 × 10^−4^	1.21 × 10^−4^	6.86 × 10^−5^	4.22 × 10^−5^	0.00 × 10^0^	0.00 × 10^0^	0.00 × 10^0^
*Clostridium leptum*	4.25 × 10^−2^	1.56 × 10^−3^	1.51 × 10^−3^	1.29 × 10^−3^	1.38 × 10^−3^	1.04 × 10^−3^	6.93 × 10^−4^	2.28 × 10^−4^
**Alignment**	**FDR P**	**5 Gb**	**3 Gb**	**1 Gb**	**0.75 Gb**	**0.5 Gb**	**0.25 Gb**	**0.1 Gb**
*C. spiroforme*	1.00	2.45 × 10^−3^	2.45 × 10^−3^	2.45 × 10^−3^	2.46 × 10^−3^	2.46 × 10^−3^	2.46 × 10^−3^	2.48 × 10^−3^
*L. lactis*	1.00	3.79 × 10^−4^	3.79 × 10^−4^	3.78 × 10^−4^	3.78 × 10^−4^	3.78 × 10^−4^	3.62 × 10^−4^	3.84 × 10^−4^
*R. inulinivorans*	1.00	8.26 × 10^−4^	8.27 × 10^−4^	8.17 × 10^−4^	8.15 × 10^−4^	8.16 × 10^−4^	8.31 × 10^−4^	8.27 × 10^−4^
*L.* bacterium 3 1 46FAA	1.00	1.71 × 10^−4^	1.70 × 10^−4^	1.71 × 10^−4^	1.70 × 10^−4^	1.72 × 10^−4^	1.72 × 10^−4^	1.69 × 10^−4^
*A. colihominis*	1.00	5.11 × 10^−4^	5.08 × 10^−4^	5.05 × 10^−4^	5.02 × 10^−4^	5.00 × 10^−4^	5.04 × 10^−4^	4.97 × 10^−4^
*E. lenta*	1.00	8.92 × 10^−4^	8.93 × 10^−4^	8.95 × 10^−4^	8.95 × 10^−4^	8.90 × 10^−4^	8.96 × 10^−4^	9.18 × 10^−4^
*Clostridium bolteae*	1.00	1.83 × 10^−4^	1.83 × 10^−4^	1.83 × 10^−4^	1.83 × 10^−4^	1.81 × 10^−4^	1.83 × 10^−4^	1.81 × 10^−4^
*S. parasanguinis*	1.00	1.90 × 10^−4^	1.90 × 10^−4^	1.91 × 10^−4^	1.91 × 10^−4^	1.95 × 10^−4^	1.80 × 10^−4^	1.79 × 10^−4^
*Lachnospiraceae* bacterium 7 1 58FAA	1.00	2.77 × 10^−4^	2.78 × 10^−4^	2.79 × 10^−4^	2.80 × 10^−4^	2.84 × 10^−4^	2.84 × 10^−4^	2.78 × 10^−4^
*H. filiformis*	1.00	3.08 × 10^−4^	3.08 × 10^−4^	3.11 × 10^−4^	3.11 × 10^−4^	3.14 × 10^−4^	3.10 × 10^−4^	3.19 × 10^−4^
*E. eligens*	1.00	7.07 × 10^−4^	7.04 × 10^−4^	7.08 × 10^−4^	7.12 × 10^−4^	7.19 × 10^−4^	7.19 × 10^−4^	7.28 × 10^−4^
*Clostridium leptum*	1.00	1.25 × 10^−3^	1.25 × 10^−3^	1.25 × 10^−3^	1.25 × 10^−3^	1.26 × 10^−3^	1.28 × 10^−3^	1.29 × 10^−3^

**Table 2:** Summary of shotgun metagenomic sequencing results at various sequencing depths and annotation with marker gene mapping (MetaPhlAn2) and alignment (BURST) at genus and species levels. Genus level results were compared to 16S information. Table also shows summary of pathway information annotated using UniProt and MetaCyc, as well as virome information at various sequencing depths.

Bacterial Genera	**Marker Gene-Mapping**
	α-diversity: Number of identified genera is slightly lower compared to alignment and 16S methods. May require > 0.5 Gb to capture diversity similar to 5 Gb. β-diversity: Grouping of the data is subject- and not sequencing-depth-based. Taxonomy/Abundance: Detection may depend on relative abundance (i.e., low abundant bacteria may not be detected at lower sequencing depths).
	**Alignment**
	α-diversity: Number of identified genera is higher compared to marker gene-mapping and 16S methods. May require > 0.25 Gb to capture diversity similar to 5 Gb. β-diversity: Grouping of the data is subject- and not sequencing-depth-based. Taxonomy/Abundance: Detection may not depend on relative abundance (i.e., bacteria in low abundances may be detected at lower sequencing depths).
	**16S**
	α-diversity: Number of identified genera is slightly higher than marker gene-mapping, but lower than alignment approach with additional filtering of taxa present at relative abundances < 0.01%. β-diversity: Data group more closely to alignment data. Taxonomy/Abundance: Genus level resolution achieved.
Bacterial Species	**Marker gene-Mapping**
	α-diversity: Number of identified species is slightly lower compared to alignment method. May require > 0.5 Gb to capture diversity similar to 5 Gb. β-diversity: Grouping of the data is subject- and not sequencing-depth-based. Taxonomy/Abundance: Detection may depend on relative abundance (i.e., low abundant bacteria may not be detected at lower sequencing depths).
	**Alignment**
	α-diversity: Number of identified species is slightly higher compared to marker gene-mapping method. May require > 0.5 Gb to capture diversity similar to 5 Gb. β-diversity: Grouping of the data is subject- and not sequencing-depth-based. Taxonomy/Abundance: Detection may not depend on relative abundance (i.e., bacteria in low abundances are detected at lower sequencing depths). Alignment to closely related taxa may result in false positives.
	**16S**
	One variable region (i.e., V4) did not discriminate species level. Multiple variable regions may be required for species level resolution.
Pathways	**UniProt**
	α-diversity: May require > 0.75 Gb to capture diversity similar to 5 Gb. Taxonomy/Abundance: Relative abundances across the sequencing depths are category-specific.
	**MetaCyc**
	α-diversity: May require > 0.75 Gb to capture diversity similar to 5 Gb. Taxonomy/Abundance: Relative abundances across the sequencing depths are category-specific.
Virome	α-diversity: May require > 5 Gb to capture diversity if no concentration/filtration method has been applied. Taxonomy/Abundance: Virus taxa are highly individualized.

## Supplementary Materials

The following are available online at https://www.mdpi.com/2073-4425/11/11/1380/s1. Dataset 1: Alpha Diversity Values WGS and 16S Genus-Species. Dataset 2: Statistics Alpha Diversity Values WGS and 16S Species. Dataset 3: Genus level classification marker gene vs. alignment vs. 16S_0.01%. Dataset 4: Group_Significance_Genera_0.01%. Dataset 5: Species_level classification marker gene vs. alignment_0.01%. Dataset 6: Group_Significance_Species. Dataset 7: Mock Community Composition_0.01%. Dataset 8: Virome. Dataset 9: Pathways UniProt. Dataset 10: Group Significance Pathways UniProt. Dataset 11: Pathways MetaCyc. Dataset 12: Group Significance Pathways MetaCyc. Figure S1: Mock Community_0.01%. Table S1. Read Statistics.
