# Supplementary material for: Metagenomic Information Recovery from Human Stool Samples Is Influenced by Sequencing Depth and Profiling Method
Source: Genes (Basel). 2020 Nov 21;11(11):1380. doi: 10.3390/genes11111380 (PMC7700633; doi:10.3390/genes11111380)
Supplement: Supplementary file 1 [file genes-11-01380-s001.zip › Supplementary Table S1.docx]

**Supplementary Table S1:** Read Statistics

| Sample | Average Sequence  Length | Total number  of sequences | Percent failed sequences | GC percent |
| --- | --- | --- | --- | --- |
| MSA1001_Read_1 | 147 | 28,738,506 | 27.27 | 53 |
| MSA1001_Read_2 | 146 | 28,738,506 | 27.27 | 53 |
| T1995_Read_1 | 148 | 30,168,684 | 27.27 | 42 |
| T1995_Read_2 | 147 | 30,168,684 | 27.27 | 42 |
| T3714_Read_1 | 148 | 31,342,712 | 9.09 | 42 |
| T3714_Read_2 | 146 | 31,342,712 | 9.09 | 42 |
| T3806_Read_1 | 148 | 23,401,270 | 9.09 | 45 |
| T3806_Read_2 | 146 | 23,401,270 | 9.09 | 45 |
| T4669_Read_1 | 148 | 17,301,936 | 9.09 | 46 |
| T4669_Read_2 | 146 | 17,301,936 | 9.09 | 46 |
| T5630_Read_1 | 148 | 31,000,131 | 9.09 | 45 |
| T5630_Read_2 | 147 | 31,000,131 | 9.09 | 45 |
| T5631_Read_1 | 148 | 23,273,845 | 9.09 | 44 |
| T5631_Read_2 | 147 | 23,273,845 | 9.09 | 44 |
| T5632_Read_1 | 148 | 26,655,115 | 9.09 | 49 |
| T5632_Read_2 | 147 | 26,655,115 | 9.09 | 49 |
| T6001_Read_1 | 148 | 20,671,316 | 9.09 | 47 |
| T6001_Read_2 | 146 | 20,671,316 | 9.09 | 47 |
| T6383_Read_1 | 148 | 18,668,053 | 9.09 | 43 |
| T6383_Read_2 | 146 | 18,668,053 | 9.09 | 44 |
| T6384_Read_1 | 148 | 32,431,559 | 9.09 | 47 |
| T6384_Read_2 | 146 | 32,431,559 | 18.18 | 47 |
