# Supplementary figures and images for: Metagenomic Information Recovery from Human Stool Samples Is Influenced by Sequencing Depth and Profiling Method
Source: Genes (Basel). 2020 Nov 21;11(11):1380. doi: 10.3390/genes11111380 (PMC7700633; doi:10.3390/genes11111380)

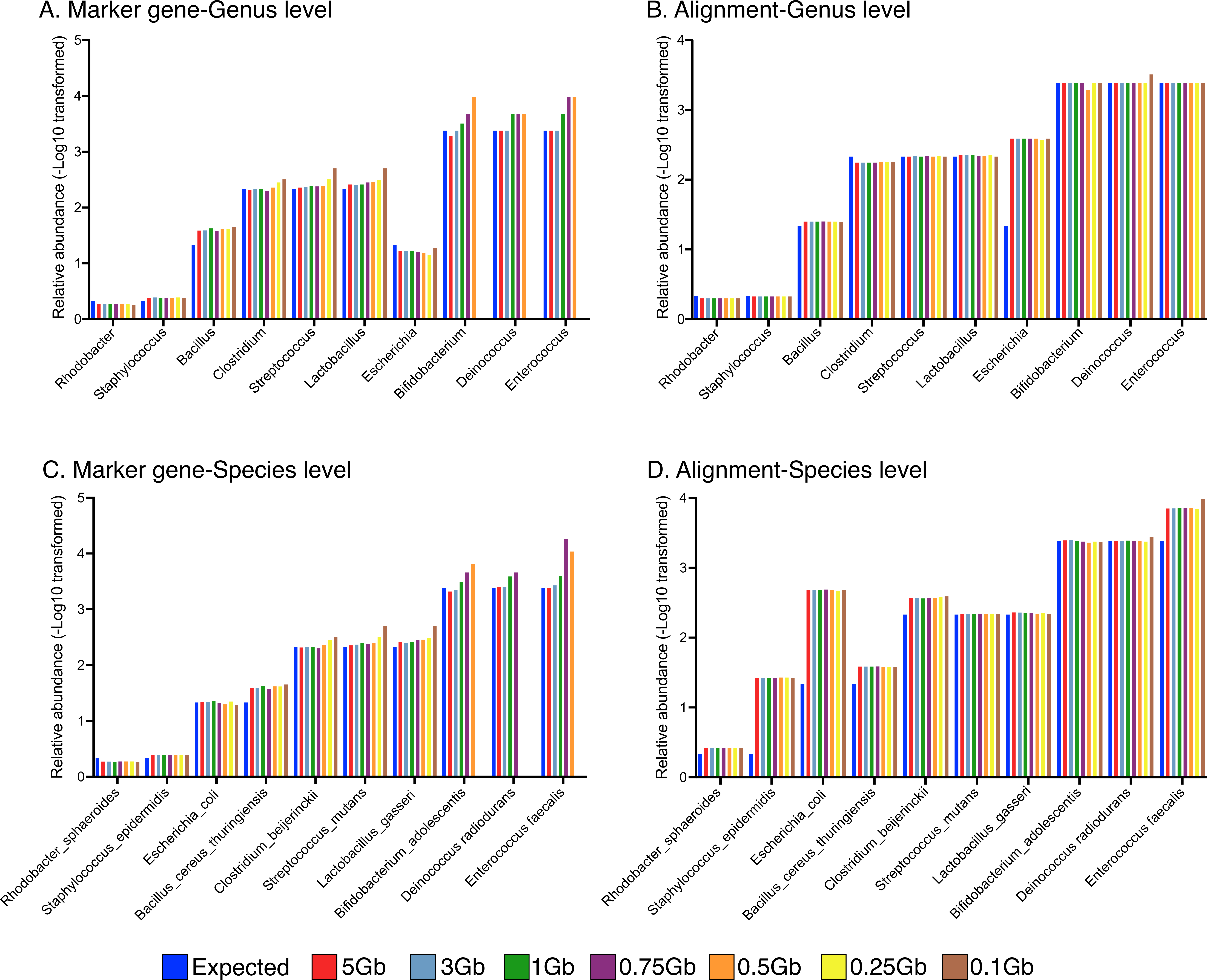

Supplement: Supplementary file 1 [file genes-11-01380-s001.zip › Supplementary Figure 1_Mock Community_0.01%.tif]
